# Political legitimacy after the pits: Corruption narratives and labour power in a former coalmining town in England

**DOI:** 10.1111/1468-4446.13169

**Published:** 2024-11-18

**Authors:** Sacha Hilhorst

**Affiliations:** ^1^ London School of Economics and Political Science London UK

**Keywords:** corruption, deindustrialisation, legitimacy, political ethnography, power

## Abstract

This article examines the erosion of political legitimacy in ex‐mining towns in England. Political sociologists and political scientists have long taken an interest in the politics of coalmining areas, which were characterised by high strike rates and militant left values. More recently, the question of legitimacy in these areas has resurfaced, as now‐deindustrialised pit towns register unusually high levels of political discontent and disengagement compared to areas with similar economic and demographic profiles. In interviews and group discussions with 93 residents of the former mining town of Mansfield, England, I find that many express ideas that profoundly challenge the system of representative democracy in its current form, with almost one in three participants understanding politics primarily through the frame of corruption. Drawing on an emergent literature which casts corruption talk as a moralised discourse of political in/exclusion, I argue that the corruption frame is best understood as the inversion of a now‐defunct symbolic economy. As workers in pit towns no longer received the same tokens of care from their representatives, reflecting their reduced power, many came to understand the political system as corrupt and illegitimate.

## 1 INTRODUCTION

In *Some Social Requisites of Democracy*, a seminal paper remembered mainly for the connection it posits between capitalist economic development and democracy (Archer, 2010), the political sociologist Seymour Martin Lipset also makes a number of pronouncements about coalmining regions (Lipset, 1959b). Lipset stresses the importance of legitimacy and democratic values for the stability of a democratic polity. Coalmining towns, he argues, tend to be heavily left‐wing and militant because of their comparative isolation, (cf. the Kerr‐Siegel model, see Kerr & Siegel, 1954) which presents a danger to democratic virtues. The dense and uniform civic networks developed in isolated, working‐class locales may inhibit workers' willingness to consent to the compromises required to maintain a democracy. As economies develop, he notes, the relative or absolute share of jobs in industries such as coalmining is liable to decline, which will be beneficial to democracy. Thus, Lipset argues, the factors driving economic development also drive “the historic institutionalisation of the values of legitimacy and tolerance” (Lipset, 1959b, p. 98).

With the benefit of more than half a century of hindsight, it has become clear that the disappearance of coalmining jobs and the development of more diverse economies and a more varied civic life in erstwhile coalmining regions has not coincided with a healthier democratic culture. Quite to the contrary, quantitative studies find that former coalmining regions register low levels of political trust, a low sense of political efficacy and a low rate of voter turnout compared to other areas with similar economic and demographic profiles (Abreu & Jones, 2021). Like other post‐industrial areas, historic English coalmining constituencies tended to vote heavily in favour of the Leave campaign in the 2016 EU Referendum and industrial heritage appears to have played a statistically significant role (Langella & Manning, 2016). Scholars have described these patterns as ‘geographies of discontent’ (De Ruyter et al., 2021; Hendrickson et al., 2018), indicative of a place‐based political cleavage (Ford & Jennings, 2020). In the elections of 2017 and 2019, many of these constituencies were part of the fall of the so‐called Red Wall, before swinging back to Labour in 2024 on a sharply reduced turnout (Curtice, 2024). In the aftermath of the loss of the coalmining industry, as its attendant institutions and infrastructures slowly decline in what has been called the ‘half‐life of deindustrialization’ (Linkon, 2018), many citizens in England's coal country express profound challenges to the legitimacy of the system of representative democracy in its present form.

If there is indeed a deficit of legitimacy in former coalmining areas, how, if at all, does this relate to these areas' industrial heritage? Because deindustrialisation is a multifaceted process with intertwining economic, social and affective dimensions (Emery, 2019; Nettleingham, 2019; Strangleman, 2017), it can be hard to tease out its various strands to identify the relevant processes. Although scholars have provided valuable descriptions and analyses of the political shifts in former industrial areas, the mechanism through which a local experience of deindustrialisation might translate into citizens' political dispositions remains underspecified. In this paper, I present a political ethnography of the town of Mansfield, which is often described as the archetypical ‘left behind’ town (Goodwin & Heath, 2016; Berry, 2017; similarly in Gartzou‐Katsouyanni et al., 2018; Gartzou‐Katsouyanni et al., 2021). Based on ethnographic fieldwork, including formal interviews with 71 local residents and their representatives and informal roundtable discussions with a further 22 residents, I find that a sizeable share of interviewees understood politics primarily through the frame of *corruption*. The corruption frame formed both a powerful challenge to the perceived legitimacy of the political system and often a motive for acquiescence in the face of ill‐defined, overwhelmingly powerful forces.

The connection between the corruption frame and the historical trajectory of the area can be understood within the traditional parameters of political sociology as laid out by Lipset, drawing on some of the same strands: legitimacy, economic change, and geography. The analysis, however, is almost exactly reversed. Where pluralist scholarship emphasised the isolation of mining towns, more recent work has pointed to their connectedness instead. British miners were able to mobilise and exercise considerable political power because they controlled chokepoints in the production and distribution of coal, which they were able to leverage from the late 19^th^ century onwards to win social and democratic rights (Mitchell, 2009). Unions, however imperfect, offered the rank‐and‐file a foothold in democratic politics.

The disappearance of the coal industry has refigured not just the economic prospects of residents of mining areas, but also the social, cultural and political amenities available to them. Having been disempowered by the deindustrialisation process, citizens were unable to prevent the loss of the civic amenities won by the labour movement. Sports pitches became newbuild housing, welfares were shuttered (Emery, 2020) and access to medical facilities was reduced. Because these facilities had historically been narrated in moral terms—a ‘token of care’ provided by political elites in recognition of miners' hard work—their disappearance carried a moral weight. I argue therefore that in light of a history of moralistic legitimations of authority, the charge of corruption is particularly powerful as its inversion, making politicians into moral outcasts. Dovetailing with wider social and political trends, such as the slackening of the connection between working‐class voters and the Labour Party (Evans & Tilley, 2017; Sobolewska & Ford, 2020, chapter 5) this helps us understand why processes of political legitimation are especially fraught in former mining areas.

In the sections that follow, I lay out a definition of legitimacy and touch on debates on its status in mining areas. I proceed to present the results of a thematic analysis of in‐depth interviews with the residents of Mansfield, UK. In the discussion section, I draw on work in sociology, political science and critical geography to connect the views of the interviewees to the economic shifts in the town around them, identifying a mechanism through which broad economic shifts translate into a distinctive and highly dismissive outlook on politics. In short, the demise of social and political infrastructures (leisure centres, well‐maintained public spaces, strong connections to political representatives), which had been won by industrial workers in positions of power and which were historically narrated in highly moralised terms, has come to be interpreted by many citizens as corruption, informing a challenge to the legitimacy of the political system as a whole.

## 2 POLITICAL LEGITIMACY AND COALMINING AREAS

In his argument on coal regions and political legitimacy, Lipset posits that the stability of a given democratic order depends on its ability to legitimate itself, that is, its capacity ‘to engender and maintain the belief that existing political institutions are the most appropriate or proper ones for the society’ (Lipset, 1959b, p. 86). This builds on Max Weber's argument that legitimacy is essential for the long‐run stability of authority or rule (*Herrschaft*) (Sennett, 1993; Weber, 1978). Weber argued that those in power may be able to sustain their rule through coercion, habit, or material interest in the short run, but in the long run, social orders in which the dominant fail to legitimate their domination become inherently unstable (Weber, 1978). ‘External’ factors such as coercion or private interest cannot guarantee the longer‐term acquiescence of the dominated, since people are motivated not only by instrumentally‐rational assessments of the outcome of a particular course of action, but also by a sense of its intrinsic value (cf. Marquez, 2016, p. 21).

Drawing on the Weberian definition, I understand legitimacy to involve the beliefs which lead citizens to consent to be ruled by another, even in the absence of self‐interest (cf. McQuarrie, 2013; Sennett, 1993). These beliefs can be understood, as per recent scholarship, to be part of a relationship between citizens and those who rule them, dependent on citizens' norms, expectations and perceptions (Schoon, 2022). The attention to expectations and norms for assent serves to distinguish legitimacy from related constructs such as political trust (Mckay et al., 2021; Van der Meer, 2017), even if political trust can condition legitimacy perceptions. For example, mistrustful citizens are more likely to perceive politicians' actions as corrupt (Wroe et al., 2013), which in turn affects legitimacy (Erlingsson et al., 2016; Linde & Erlingsson, 2013; Seligson, 2002). Furthermore, citizens' relation to the object of legitimacy may be mediated by various institutions (Schoon, 2022). In the case of coal regions, workers' attitude towards political authorities may be mediated by their membership in a militant union, which could increase their willingness to consent to the existing political system by giving them a stake in its reproduction, or undermine it as political alternatives become imaginable.

Present‐day concerns over the (presumed) flourishing of illiberal values in (ex‐)industrial locales (e.g., Hochschild, 2024), centred on angry voters in ‘left behind’ places (see Emery, 2023), are prefigured by an earlier generation of scholarship which read mining communities as prone to ‘extremism’ (Lipset, 1959a, 1959b, p. 96). To the pluralists, mining communities demonstrated the dangers of densely networked, homogenous political cultures, reflecting an abiding concern with the social structures which might inculcate tolerance and moderation. Drawing on what became known as the Kerr‐Siegel hypothesis (Kerr & Siegel, 1954), Lipset pointed to miners' relative isolation to explain their militancy (Lipset, 1959b), as miners in remote pit villages shared social lives as well as workplace grievances. While it is true British miners went on strike far more frequently than workers in other industries (Church et al., 1991), the presumed remoteness and homogeneity of mining communities underplayed differences between and within coalfields. This informed a variety of tropes which cast miners as traditionalist and communitarian, with mining communities representing for some scholars a valuable repository of ‘authentic’ working‐class culture and for others a seedbed of antidemocratic values (Strangleman, 2018, pp. 20–21; Arnold, 2024, pp. 288–290).

The Kerr‐Siegel hypothesis would be challenged by scholars in later decades (Church et al., 1991; Rimlinger, 1959; Scott & Marshall, 2009), for overlooking among other things the power that workers derived from their ability to control the flow of carbon energy (Mitchell, 2009). In coal‐dependent, industrialised countries, mining towns found themselves at crucial points in the nation's energy supply, which empowered miners and workers at other chokepoints of energy production and distribution, such as railway, port and power station workers, to make radical social and democratic demands (Mitchell, 2009). Isolation mattered insofar as it bolstered this logistical power. As Timothy Mitchell argues in his Foucauldian account of legitimacy, the nation's coal dependency strengthened claims for democratisation at home (Mitchell, 2011), while also fostering imperialism abroad (Mitchell, 2011; in particular, the shift to coal power resulted directly in the conquest of new colonies to function as coaling stations. See Khalili, 2020).

By the mid‐1990s, readings of miners as affluent workers or dangerous revolutionaries were supplanted by accounts focused on trauma, community and loss (Arnold, 2024, p. 290). The academic literature has stressed that the legacies of industry continue to be felt long after physical structures have disappeared (Strangleman, 2018), with deindustrialisation ‘an active and significant part of the present’ (Linkon, 2018, p. 2). These ghosts, spectres and half‐lives of deindustrialisation now appear to be haunting British politics, with former coalfield areas identified as a driving force behind political upsets of recent years. Some scholars have argued that post‐industrial areas are ‘in revolt’ because they have suffered neglect and disinvestment from the national government (Hudson & Beynon, 2021), having been made into ‘places that don't matter’ (Rodríguez‐Pose, 2018). This has brought renewed attention to the politics of (post‐)industrial areas, with expressions of discontent now read not as a powerful social faction flexing its muscles, but as the rear‐guard action of a declining class.

Thus questions of power and legitimacy loom over the politics of ex‐industrial England, as it becomes increasingly clear that loss of power and status has produced a backlash. This is not primarily about economic hardship per se, as recent expressions of political discontent do not map onto local deprivation or individual incomes so neatly (Abreu & Jones, 2021; Abreu & Öner, 2020). Scholarship on the politics of ex‐industrial areas has pointed instead to the role of status loss and (relative) decline, emphasising emotions such as pain (Silva, 2019) and shame (Hochschild, 2024) as well as the psychological effects of status loss among working‐class citizens more broadly (Gest et al., 2018; Gidron & Hall, 2017; Kurer & Staalduinen, 2022). As their home towns become increasingly peripheral (Olivas Osuna et al., 2021), it appears that residents of ex‐industrial towns make political judgements about fairness and representation based not just on an assessment of their own position but also of the places they live in (similar in Cramer, 2016a), which can fuel resentment (Cramer, 2016b).

For all their merits, however, these accounts have engaged little with the changing shape of the socio‐political institutions which mediate between structural position and (political) subjectivity. The structural position of the miners as producers of a strategically important resource interacted with pre‐existing political cultures which varied by coalfield (Beynon & Austrin, 1994) and found expression in institutions such as clubs, unions and local Labour chapters which organised social life and political subjectivities in distinctive ways. As the literature on the half‐life of deindustrialisation attests to, the transformation or loss of such legacies is not straightforward or linear, as remnants of industrial‐era structures, discourses and subjectivities interact with a novel political economy. This long‐term process of institutional change can help us understand the nature and timing of political shifts (Pacewicz, 2016a, 2016b). Here, I contribute to the literature by specifying one mechanism through which the half‐life of deindustrialisation shapes political discontent, restoring ethnographic specificity to a type of community which has too often been rendered as archetype or foil (Strangleman, 2018).

## 3 METHODOLOGY AND CASE SELECTION

Political ethnography as a methodological tradition has proven apt at illuminating people's everyday engagements with politics (Baiocchi & Connor, 2008; Benzecry & Baiocchi, 2017; Tilly, 2006). Through thick description, it can offer insight into the way larger forces become social realities and the way political processes take shape in concrete settings (Baiocchi & Connor, 2008; Tilly, 2006). Methodological choices in the subfield reflect an ambition to not define the political a priori. After a broad exploratory phase, I employed interviews and participant observation in order to capture citizens' narration of the political bond as well as the way these narrations and identifications were negotiated within local interactions and shaped by the institutions around them.

Mansfield frequently appears in the popular and scholarly literature as the archetypical ‘left behind’ town (Goodwin & Heath, 2016; Berry, 2017; similarly in Gartzou‐Katsouyanni et al., 2018; Gartzou‐Katsouyanni et al., 2021). At 70.9%, the constituency recorded one of the highest vote shares for Leave in the 2016 EU Referendum, giving it the 15^th^ highest Brexit vote in the country. When the seat fell to the Conservative Party in 2017, with Labour losing the seat for the first time in almost a century, it helped to inform the Conservatives' ‘Red Wall’ strategy, which would soon see neighbouring constituencies such as Ashfield and Bolsover captured by the Conservative Party as well. Since the 2024 election, the Labour Party has held the Mansfield constituency once again, having won it back on a depressed turnout of 55.8%.

As has been noted of other coalfields (Beynon & Austrin, 1994), the political culture, anchored in a dense set of workers' institutions built up by the labour movement (Taylor, 1972), was traditionally staunchly Labour but not left‐wing (Gibbon, 1988).^1^ A notable example is the 1984‐85 miners' strike. Many Nottinghamshire men defied the call to strike, joining the breakaway Union of Democratic Mineworkers instead. Mansfield miners often believed they had jobs for life and only few Nottinghamshire miners persisted striking until the end (Beynon, 1985; Emery, 2018; Gibbon, 1988; Samuel, Bloomfield and Boanas, 1986). Local NUM banners commemorating the strike read ‘so few, so strong’. These histories nuance the image of the stalwart Labour heartland, as the meaning and political implication of the area's mining tradition has long been subject to widely differing interpretations.

Although the town is now associated with post‐industrial decline, it was once known for its high incomes and thriving manufacturing base. The town lies on top of the rich North Nottinghamshire coalfield, which was regarded as the jewel in the crown of the National Coal Board (Emery, 2018). Men often worked in the many pits which ringed the centre, while many women found employment in shops, offices and especially the textile factories in and around town. The late 1980s saw an acceleration of pit closures, with the final mine in the county closing in 2015. The 1990s and 2000s subsequently saw the disappearance of the local textile industry, as major retailers such as Marks and Spencer offshored their supply chains. In regeneration efforts, councils and development agencies presented locals as willing to do hard work at odd hours for comparatively low wages (Strangleman et al., 1999). At present, many locals are employed by the local hospital, large supermarkets and care homes, often for poor pay, while other local residents commute to larger, more affluent towns and cities in the region. Being centrally placed within the country, the town has become a hub for low‐paid distribution centres and logistics work, with Sports Direct and Amazon major local employers (Done‐Johnson, 2021).

After several months of intermittent visits and a long break during the height of the COVID‐19 pandemic, I spent four months in late 2021 working in the field, living locally and conducting participant observation in community venues. Several locals acted as my guides. The generous help of my guides was all the more invaluable because I was noticeably an outsider to the community, in a place where outsiders can be met with mistrust. I was also noticeably an immigrant and although I have lived in the UK for many years, interviewees often asked me where my accent was from. As a stranger to the town, I am certain to have missed nuances and contexts. An outsider status, however, can also be an advantage (Sana et al., 2016), as interviewees spoke with remarkable candour about intensely private life events.

The sample was balanced in gender and age. Only two of my respondents were black, which is in line with the local area, but places limits on the analysis. Immigrants were somewhat underrepresented compared to the local demographic profile, in part because some of the more precariously employed immigrants I spoke to were hesitant to be interviewed for fear of workplace repercussions. In terms of subjective social class, the vast majority described themselves as working class, with many having grown up in pit families where a father, an uncle or at least a grandfather worked down the mine. 18 participants had been miners themselves. These interviewees were concentrated in the group discussions, although some agreed to longer sit‐down interviews as well. All of these men were now retired, with the exception of two who continued to run small businesses. Among those interviewees presently in work, employment varied from manual work, for example, as a cleaner at the local Centre Parcs, to lower‐level administrative jobs, retail, social care, as well as work for local schools, the hospital or the council. The small number of interviewees who did not identify as working class included one person who worked with local libraries, someone with a well‐paid job at a large housing association and some local businessmen.

My predominant field sites were the local football club, a café, the pub and a community gardens/allotment. When invited, I also joined for birthday parties, festivities and a funeral.

Interviews were conducted in people's homes or in local cafés where the owners facilitated a suitable space for me. The interviews were designed to initially elicit processual accounts, including questions about people's biographies, their relation to their town and the role politics had played in their lives, if any. As the conversation progressed, I sought to understand the norms, expectations and perceptions people brought to bear on politics as well as the factors shaping those norms, expectations and perceptions. Which salient contexts did they reference? Which interactions and contexts shaped them? How did they *move* in political settings, broadly conceived?

The resulting data included four months' worth of field notes, transcripts for formal interviews with 71 people and detailed notes for more informal roundtable group discussions with a further 22 residents in small groups. The average length for the formal interviews was approximately 90 min and the informal group discussions were a minimum of 45 min (shorter discussions were included in the general field notes). These were analysed using an abductive analytical strategy (Timmermans & Tavory, 2012). Throughout the process, I compared my findings with literatures on political discontent in former industrial regions. During the fieldwork and in the subsequent analysis I consistently wrote memos, in an iterative process of coding and memo‐writing which included rounds of looking for confirming cases as well as rounds devoted to finding disconfirming evidence.

## 4 A MORALISED REJECTION OF POLITICS

**FIGURE 1:**
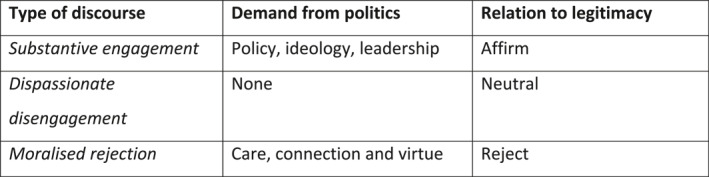
Discourses about politics and their relation to legitimacy. Drawn from a thematic analysis of Mansfield interview data, 2020–2022.

Interviewees differed widely in their willingness to engage substantively with the political system. Relations to the existing system of representative democracy could be broadly categorised as ranging from *substantive engagement* to *dispassionate disengagement* to *moralised rejection* (see Figure 1). In tracing their own relationship to politics, some engaged substantively, speaking about values (Surridge, 2021), ideology, partisan identification, and perceptions of competence and leadership (Clarke et al., 2011), congruent with the political science literature on how citizens make political judgements. Others were dispassionately disengaged and preferred not to express a view on political matters, viewing the political system neither positively nor negatively. One interviewee said she had avoided watching the news and engaging with politics in any way. ‘I've been pregnant pretty much all year, it seems like’, she said, ‘and I just felt like I just needed positive things’. In line with Nina Eliasoph's portrait of apathy in civic life, these interviewees regarded politics as unpleasant or uninteresting and preferred to stay away from ‘divisive’ or ‘boring’ political issues (Eliasoph, 1998). Some scholars have argued this seeming disinterest among working‐class citizens reflects the erosion of class as the organising frame of politics (Manning & Holmes, 2013, 2014).

A third group offered a moralised rejection of politics. Although this group was superficially similar to the disengaged group in that they too felt politics had little to offer, they expressed harsh moral judgements of politics and politicians. In particular, many expressed the view that politicians and the political system at large were corrupt. This was meant both literally and metaphorically: interviewees used the term ‘corruption’ to allege that politicians were caught up in illicit financial ties (talking about politicians taking ‘backhanders’, ‘lining their pockets’, ‘money in brown paper envelopes’), but simultaneously invoked corruption to express a broader sense that politicians were moral reprobates. Corruption then could simultaneously express an allegation of criminal wrongdoing and a broader sense of moral decay and failing structures of representation. Participants would occasionally move between different discourses on politics, but in general those who expressed the view that politics was corrupt also felt that the political system was broken or somehow in a state of decay. The allegation of corruption represented a profound challenge to the authority of those politicians who were deemed corrupt (which was, for many interviewees, all politicians), as well as to the legitimacy of the political system at large.

In this paper, I focus on those interviewees, amounting to roughly one in three residents in my sample, who offered a specific moralised rejection of the political system, understanding politics in large part through the frame of corruption. Understood as a moralised discourse regarding ‘the abuse of entrusted power and resulting social decay’, the corruption of the present was implicitly contrasted with some uncorrupted past or place (Doshi & Ranganathan, 2019, pp. 2–3). The perception of corruption was connected to an unmet expectation of care and the disavowal of social ties. Although many interviewees were proud to make up their own minds in elections—often drawing a contrast with an older generation which ‘mindlessly’ voted for the Labour Party—in moving away from the dogmatism of their elders, many had arrived at a stance of generalised cynicism, to the point that they did not believe, or professed to not believe, that *any* politicians were *ever* sincere. Regardless of their ideological commitments or policy achievements, politicians were seen as corrupt. They were considered illegitimate representatives and outcrops of a broken system.

### 4.1 ‘Lining their pockets’: Narrating corruption

In my second month living in Mansfield, one of my local guides introduced me to Millie, a local care worker. Politically, Millie felt disenfranchised. Her father had been a miner, whose politics in Millie's recollection mostly revolved around a passionate hatred of Margaret Thatcher. She recalls being taken on marches to protest pit closures. Millie mistrusted not just Thatcher, but all politicians, as well as virtually all media organisations.

Millie I didn't understand politics, I still don't really understand politics and I'd rather not watch. I don't watch the BBC anymore. I don't believe they tell the truth. I believe it's propaganda, it's there to make people sway.

SH To make people what?

Millie To sway to how they're thinking. I don't think half of it's true. There is always hidden agendas. That goes back to the Margaret Thatcher days, hidden agendas around the pits shutting. Like, they put something out there to make us believe but meanwhile they're covering up something else. [That's] the way I've seen it as a child and me growing up in my adult life.

Millie usually voted for a third party candidate. She felt politicians looked down at regular people like her. ‘Don't like Labour much, don't like the Tories, at all. They come in, ‘You will do as we say.’ Don't have much of a chance of standing up to them’. She expected escalating disorder: ‘I've been predicting there will be riots soon, I've been saying that for a long time. And I think all this [the COVID pandemic] is to privatise NHS to be honest’. Politicians represented the inverse of the values of honesty, solidarity and hard work which she tried to live by. ‘I don't agree with Labour, I think they're liars. And I think the Tories are liars. And it's always the one or the other, isn't it’. When asked what might motivate politicians to lie, she answered swiftly: ‘Money. It's always money. Money and greed. You're not telling me they're not having their pockets lined’.

This framing of politics was connected for Millie to her own struggle to make ends meet and her sense of local decline. Millie had been raised with a firm work ethic. Her grandmother, at age 70, worked a shift the night before she died. ‘She would always say, if I give up work, I'll die. Or retire, I die. And she ended up dying anyway’. Millie grew up in Mansfield in a landscape defined by the pits, not just through the headstocks that marked the view but also the welfares and sports pitches. It bothered her that so many of the colliery pitches had now been sold off or made into housing.

Millie Do you know what I mean? It's the community.

SH And there's that that infrastructure that that was that community infrastructure being…

Millie Took away. What do you want kids to do, they can't play football, they can't play hockey. […] I used to be part of the pit band, the brass band. When the pits were about you used to have colliery bands. I used to play with Welbeck Colliery Band, Thoresby Colliery. When the pits started closing they lost funding and stuff. I mean, it's still about don't get me wrong. But it's not as rife as it used to be. […] I got a neighbour who also worked down the pit, but his daughter played for the brass band, they brought me into it that way. So you know. That's all gone. And then school, from school actually used to do a music school on Saturday morning for 11–18 year olds. Yeah, that disappeared, the Sure Starts disappeared. Everything's just horrible because I'm watching… My kids don't understand it because they never had it. But it hurts me, because I am like, what am I to do.

In Millie's narration, the sense that things had been taken away went hand in hand with a sense of civic decay and a mistrust of politics.

Campaigning in Mansfield, senior Labour figures have tended to describe their candidate as a local champion and someone who ‘stands up for the community’ (Mansfield and Ashfield Chad, 2017). To many residents, this was a preposterous suggestion. ‘They sort of look after themselves, the politicians’, said Robin, a now‐retired ex‐miner. ‘They don't [do] what they say, they don't keep to what they say they're going to do. They tell lies. It shouldn't be like that’. Later, he added: ‘They're making millions, aren't they?’ These themes were echoed by other ex‐miners in one of the pubs which was central to the fieldwork. They described the previous Labour MP as ‘a waste of space’ who had done nothing to stem the loss of well‐paying jobs or the decline of the town centre, while making sure to after his own financial interests. Where the Labour Party had once been able to count upon the loyalties of Mansfield residents, many residents now saw its representatives first and foremost as self‐serving, conniving figures, whose promises and moral commitments meant little. Many expressed the feeling that ‘they [politicians] are all as bad as each other’. This meant that even (some) citizens whose economic views placed them on the left felt an aversion to the party. Politicians lie, Robin said, ‘for their own means, basically. To see if you're suckered into it or not. […] They feather their own nest’.

When I asked local councillors about the corruption talk, some described it as ‘conspiracy theories’. While it is true that interviewees sometimes repeated hoaxes, unsubstantiated local rumours and social media misinformation, these mixed with true stories about MPs' second jobs, pandemic contracts and the 2009 parliamentary expenses scandal. Nor was corruption talk limited to the socially marginal. The allegation of corruption was common across demographic categories, including among younger generations and women. One of these women was Keira, a 40‐year‐old teacher. In late 2021, we met in Keira's neatly maintained semi‐detached home in the north of Mansfield. We traced her life growing up in town in a Labour‐voting household. As she grew older, she became increasingly sceptical of the staunch Labour support among her parents's generation. People of her generation were more willing to think for themselves, she said. She had voted Conservative last time, but next time she ‘won't bother anymore’.

I feel really feel sorry for like Boris Johnson, you know? Yeah, he's the prime minister in name, isn't he? But I don't think for one minute that he makes any decisions. I think he's just the mouthpiece for it all. Just… I don't know, I just think that whoever's paying the money for whatever agenda they want him to push at that moment. It's just, it just feels like everything's just built on lies. And you never really know what's going on. But I feel that's like worldwide as well. You know, I just think corruption is just massive.

Corruption, she said, was the biggest political problem facing the country. It allowed her to make sense of her own sense of disempowerment. She believed politicians had exaggerated the extent of the threat posed by COVID. ‘Just while you know, while the rich are getting richer, it does seem like it's getting harder and harder for, for the rest of society.’

She had not encountered many examples of concrete examples of corruption herself—she was not close enough to the halls of power to be able to witness such practices, she said—but felt it was the most straightforward way of explaining why the world functioned as it did. She expected any remnants of authority to wither. Her mistrust of the media and especially the BBC ran deep.

[My husband] has probably said, you know, many a time, this kind of, “there's going to be riots.” Well, you know, like, “People, they've got to get fed up soon, Keira. They've got to get fed up of you know, hearing this and then that happening.” And it hasn't happened as far as we now. Although I am very much, I do believe the media is orchestrated to what they want you to see and what they don't want you to see. There could be rioting going off and you just don't hear about it now.

Keira was firmer than others in her belief that she was being lied to, but she was not unique. Many shared Keira's sense that there was a great rot at the heart of the system. The frame of corruption offered a distinctly moral discourse for expressing the way their representatives made them feel powerless and voiceless.

### 4.2 ‘In it for us’

On a cold morning, I arrived at a tidy semi‐detached house to speak to Michael, an ex‐miner, and his wife Mary, who had worked in the textile factories. Although Michael had fond memories of the mining industry, he knew its dangers better than most, having lost a leg in a pit accident. Now they were comfortably retired. Both Mary and Michael had recently voted Conservative, but they related to politics in very different ways. Whereas Michael was keen to discuss policies and track records, Mary was generally mistrustful. She had voted in most elections, usually Labour, before switching to the Conservatives in either 2015 or 2017, but she did not think she would be voting again.

Mary I suppose it's all the same, in't it. You know, I mean, nobody… You don't really trust a politician? Do you? I mean, come on. I mean, I don't even think they can lie straight in bed.

SH And have you always felt that way about politics?

Mary Yeah. Because I just I just think, you know, sometimes I just think, these people, they say what they think we want to hear. And I find that quite annoying. That, I find that as if they think we're thick. You know, and we don't understand what's going off. And I think they think they're placating us, and that's what I don't like, you know. We are not thick, we understand a lot better than you. […]

SH So what do you think motivates the politicians to act the way they do?

Mary Money. I just think in all it's like Michael says, You know what I mean, it's like an MP has to serve one term in Parliament, and they get pension. That's wrong. I mean, they should be doing that job for us, not for monetary gains you are going to get out of it. Because, I mean, half of them don't even [turn up]. […] [Turning to Michael] But I've said to you haven't I, I ain't voting again, I can't be arsed with it.

Here, Mary's complaint that ‘they should be doing that job for us’ echoes others' complaints, which appear frequently in the interview data. ‘They do not care.’ ‘They aren't bothered.’ ‘They are not in it for us.’ This unmet expectation of a social and moral position on the part of politicians (selfless public service, a personal investment in the lives of their constituents) that made them feel that the political system was failing not just in its outputs, but at a profound moral level.

Michael did not dispute Mary's description of politicians as conniving and self‐serving, but politicians' moral failings did not inform his general outlook on politics in the same way. His expectation of politics was not based on the moral character of politicians, but on what they had gotten done. The previous Labour MP was not all bad, he said, because he had managed to get the railway station reopened. To Mary, this was a moot point, as she judged the former MP to be of bad moral character regardless of the status of the railway station. Although Michael and Mary shared a set of grievances around the decline of the town centre and the general marginalisation of their hometown, the failure of successive politicians to turn the tide did not seem to affect Michael's general support for political representation in the same way, because their personal social and moral investment was not as important to him.

It is hypothetically possible that the perception of corruption coexists with a sense that the wider system is legitimate, perhaps because corruption talk is a way to ‘blow off steam’, or because the corruption is deemed to be sufficiently contained within well‐functioning legal and political structures. In practice, however, interviewees took the (perceived) behaviour of politicians to be indicative of the moral bankruptcy of the political system as a whole. The appeal of the corruption frame (as opposed to allegations of incompetence or misguided ideology) afforded a wide variety of complaints about the loss of political voice and the experience of economic decline a systematic thrust and moral weight. While mostly supportive of the idea of democracy, these interviewees were scathing about the actually existing system of representative democracy and those who wielded power through it.

## 5 DISCUSSION: GIFT AND OBLIGATION

‘Corruption politics is critical to the ways that everyday populations come to symbolise, imagine, and critique experiences of exploitation, neglect, and economic decline, in turn seized upon by populist figures’, write geographers Sapana Doshi and Malini Ranganathan (Doshi & Ranganathan, 2019, p. 18). The interview transcripts and field notes presented here offer a palpable sense of residents' anger at the decline of social and cultural provision in their town; the perception that politicians did not care; and the pervasiveness of the accusation of corruption. For interviewees like Mary, the belief that politicians were motivated only by personal financial gain dovetailed with a wider sense of local decline, as town centre shops had disappeared, pubs had shut and the market had shrunk. ‘There's nowt there. It's atrocious really. […] Everything went’. This echoes Millie's complaint: ‘They're just taking everything away. How does that make sense?’

The loss of social infrastructure and the changing character of representatives can be understood in structural terms, reflecting the relative economic decline of coalmining areas and changing practices of political representation (see e.g. O’Grady, 2019 on the disappearance of working‐class representatives; Sobolewska & Ford, 2020 on the professionalisation of party structures; and Lawrence, 2023 on the Labour Party becoming disembedded from local, place‐bounded institutions). Crucially, however, these were not narrated predominantly as structural shifts, which might be addressed through democratic politics, but as moral failings, which implicated political representatives and the wider system alongside them. Alongside the charge of corruption, politicians were accused of being remote from the community and failing to care.

In light of its discursive entanglement with expectations of connection and care, the charge of corruption must be read not as a request for cleanliness and propriety, that is, not as a request for impersonal politics, but as a request for recognition and sociability. Anthropologists Sarah Muir and Akhil Gupta refer to this as the contradictory desires embedded in corruption talk, ‘on one hand, for the rule of law, proceduralism, and justice, and, on the other hand, for modes of sociality, discretion, and intimacy that exceed the law’ (Muir & Gupta, 2018, p. 10). The implicit counterpart to the corrupt politician was not the impersonal administrator, but the community‐minded politician, a locally embedded servant of the public cause who was in touch with the town and emotionally invested in its residents. In describing their expectations from politics and politicians, many reached for notions of care and moral involvement from a bygone era, which they implicitly held up as a contrast with the corrupt present. Their loss of political voice and loss of access to social and cultural provision was understood to be in significant part the result of the venality of their representatives.

We might suspect that the withdrawal of social and cultural provision was read through a moralising rather than a structural lens because the provision itself, when it still existed, had been cloaked in moral language. The historical record suggests the provision of baths and leisure facilities was a straightforward reflection of the power of the labour movement, with increases in provision corresponding to periods of strike action or the threat thereof (Morgan, 1990). Nevertheless, this provision came to be narrated in moralised and quasi‐religious terms. In the words of one mid‐century politician, communal baths facilitated the miner's ‘ablutions’ and social provision for the mining community was merited because ‘no miner works harder than the British miner’ (‘Miners Welfare Bill ‐ Hansard ‐ UK Parliament’, 1952). In the words of another, ‘[I]f a man elects to earn his livelihood in the bowels of the earth producing a commodity which is so essential, when he is shut out from the health giving rays of God's sunshine, he is entitled to the best amenities when he returns to the surface’ (‘Miners Welfare Bill ‐ Hansard ‐ UK Parliament’, 1952). Social provision was perceived as a *token of care*, which allowed political representatives to prove they were on the miners' side. They offered a shared moral imaginary available to miners and their representatives alike, structured around care, community and hard work.

Understood in Bourdieusian terms, the exchange of labour power for social provision is misrecognised. It is imagined not as a negotiation between labour, capital and the state, but as a form of reciprocity, which comes to constitute a moral relation (Bourdieu, 1997; Chanial, 2010; Silber, 2009). In their industrial heyday, the citizens of towns like Mansfield received an array of concrete ‘gifts’ from their representatives. Comparatively generous local authority funding from Whitehall and concessions extracted from locally rooted industries provided funding for schemes for the welfare of local inhabitants, often directly in response to strike action. Some ethnographers of post‐industrial towns have described this as a system of gift and obligation (Pacewicz, 2016a). After nationalisation, this provision was typically provided directly by the state. People would gift their votes and in turn would receive investment in those things they cared about, such as leisure spaces and community centres, while gruelling work down the pit afforded pit families access to clubs, bands and social facilities provided by the coal board. This is not an exchange between rational economic agents, but a social tie, where the exchange is euphemised into, and subsequently misrecognised as, a social and moral relation, or even a form of care.^2^

With the Thatcher‐era reforms to local funding and the disappearance of industries which could be effectively pressured through strike action, the inhabitants of Mansfield lost their position of power and no longer received the same tokens of care. Provision was further curtailed during the austerity drive of the 2010s (Dagdeviren et al., 2019; Gray & Barford, 2018), which affected Mansfield greatly. Interviews were rife with examples of redeveloped sports pitches, shuttered social clubs and failing access to healthcare. Present‐day interviewees continue to narrate politics in profoundly moral terms, where hard work warrants certain forms of voice and care. Rather than bolster the legitimacy of the political system, however, these narrations mark politicians as reprobates and paint the present system of representative democracy as corrupt.

As the political scientist James Scott points out, forms of legitimation are also points of weakness, which can be volleyed back at the authorities (Scott, 1990). If domination has been legitimated on the basis of care, rulers become vulnerable to the allegation that they do not care. The tokens of care that were provided for much of the 20th century have been gradually withdrawn. Inhabitants ‘gifting’ their votes were not receiving the same perks in turn. If the political bond is imagined as a moral relation tied up in a mutual exchange of tokens of care, its inverse is the charge of corruption, in which gifts are bestowed only on oneself. This, I argue, is one of the pathways through which the experience of relative local decline may translate into a political disposition and specifically a disposition which is at odds with the prevailing forms of legitimation.

Critics have argued that talk of a legitimation crisis is overblown, because, they allege, if there was such a shortage of legitimacy, citizens would be far more unruly than they currently are (Marquez, 2016). It is true that most of my interviewees were politically passive. They rarely participated in protests, were not involved with any political parties or movements and although many of my older research participants had been members of a union at some point, only few were members now. If they believed, as they said they did, that politicians did not care whether they lived or died, that media organisations were deliberately lying to them, and that moneyed interests controlled the agenda, why did they not protest more? Here again the corruption frame offers an explanation. Feeling as they did that they were up against nefarious and devastatingly powerful forces, they had little hope that they could prevail against them. Corruption talk served as a powerful challenge to legitimacy as well as a motive for remaining passive. Consent, fatalism and acquiescence, while theoretically distinct, can look highly similar in practice, especially as interviewees move back and forth along different and sometimes contradictory positions over the course of an interview. Nevertheless, the delegitimising force of the corruption frame was unmistakeable.

## 6 CONCLUSION

A heterogenous body of research conducted in the aftermath of the Brexit vote has suggested that legitimation may be partially failing in England's former mining communities, as surveys register a mistrust of politics and a sense of political powerlessness. Drawing on ethnographic methods, I have argued that many residents of the town of Mansfield have come to believe that the existing system of representative democracy is riddled with corruption. Corruption talk served as a moral discourse to understand political exclusion and to diagnose the abuse of entrusted power. Many felt they had entrusted politicians with their vote and had been betrayed, as politicians failed to offer the care and consideration they ought to. This, I have argued, is one of the pathways through which the lingering effects or half‐lives of deindustrialisation can translate into a political disposition, as older modes of understanding politics collide with a novel political economy.

The account of legitimacy championed in this paper prompts a number of revisions to the classic account offered by Lipset. Pluralist political sociology of the Cold War era, which continues to inform present‐day readings of populist mobilisations, was suspicious of militant values on either side of the political spectrum (Jäger, 2017; Stavrakakis & Jäger, 2018). Writing in the *British Journal of Sociology*, Lipset himself characterised unions as undemocratic organisations (Archer, 2010; Lipset, 2010), which he suspected of fostering illiberal values and working‐class authoritarianism (Lipset, 1959a; Miller & Riessman, 1961). In the case of Mansfield, however, we can see the democratising force of the unions, which leveraged their power for wider social and democratic gains. The strength of Mansfield's labour infrastructure was not generally channelled into antidemocratic directions, but euphemised into social provision and accompanied by moral discourses of care, community and work, underpinning rather than undermining the legitimacy of the political system. Those discourses are now turned back onto the political representatives whose legitimacy they once bolstered.

Recent scholarly interventions have made the case for addressing status loss by promoting cultural outputs which reflect the lives of working‐class people in post‐industrial areas (Hochschild, 2024) and creating pathways for working‐class politicians to facilitate descriptive representation (Elsässer & Schäfer, 2022). While sympathetic, the account I have presented in this paper suggests that such interventions are not sufficient when many have come to feel politics is fundamentally corrupt. The election of working‐class MPs does not guarantee the restoration of the community‐mindedness and social connection that interviewees missed, which will require among other things the (re)creation of collective institutions through which citizens can hold politicians to account. Moreover, in the absence of investments in public provision, well‐kept commons and leisure facilities, it will be difficult for representatives to convince their constituents that they care about the people and places that elected them.

The analysis presented here has drawn attention to the mechanisms through which (loss of) power translates into a political subjectivity. Communities like Mansfield have been shorn of their structural power but not of the sense that their hard work ought to earn them certain forms of care and consideration. The public affordances of this erstwhile power have been steadily withdrawn, leaving a widespread sense of corruption in their wake. In the absence of a radical project to mobilise around, the steady erosion of legitimacy has not spiralled into an acute crisis of legitimation. Nevertheless, the failure of legitimation makes citizens available for mobilisation by heterogenous social movements, including potentially those with an illiberal thrust. In the most recent general election, almost half of eligible voters in Mansfield chose to abstain and these patterns of working‐class abstention are likely to continue or even intensify, until such a time when an upstart political movement succeeds in harnessing this anti‐political energy into a more sustained challenge to the political system.

## CONFLICT OF INTEREST STATEMENT

The author declare no conflicts of interest.

## Data Availability

The data that support the findings of this study are available on request from the corresponding author. The data are not publicly available due to privacy or ethical restrictions.

## ENDNOTES

^1^
For example, even prior to the ‘84‐85’ miners' strike, the Nottinghamshire unions tended to be more opposed to industrial action and more in favour of financial incentive schemes, which placed them on the right of the NUM (Gibbon, 1988; Krieger, 2014). Even earlier, during the famous strike of 1926, Nottinghamshire was the home of the management‐friendly Spencer union, which commanded considerable support in the region.

^2^
The classic reference for this line of thinking is of course Mauss (2002), whose thinking is channelled by Bourdieu, see Chanial (2010).
